# Is Familism a Motivator or Stressor? Relationships Between Confucian Familism, Emotional Labor, Work-Family Conflict, and Emotional Exhaustion Among Chinese Teachers

**DOI:** 10.3389/fpsyg.2021.766047

**Published:** 2021-12-02

**Authors:** Xiaoshuang Zhu, Guoxiu Tian, Hongbiao Yin, Wenjie He

**Affiliations:** ^1^College of Teacher Education, Capital Normal University, Beijing, China; ^2^Faculty of Education, Chinese University of Hong Kong, Shatin, Hong Kong SAR, China

**Keywords:** emotional labor, work-family conflict, emotional exhaustion, familism, job demands-resources model

## Abstract

To reveal the cultural effect in the job demands-resources model, this study examined how Confucian familism, emotional labor, and work-family conflict (WFC) explain the variance in teachers’ emotional exhaustion, with a focus on the mediating roles of emotional labor and WFC. With a sample of 3,312 teachers in China, the results of this study revealed that surface acting and expression of naturally felt emotion (ENFE) and WFC mediated the relationship between familism and emotional exhaustion. Moreover, familism positively predicted deep acting, ENFE, WFC, and emotional exhaustion, while negatively predicted surface acting. These findings suggest that Confucian familism may play the dual role of motivator and stressor for Chinese teachers’ emotional labor and well-being. This study contributes to the job demands-resources theory by revealing the important role of cultural traditions and provides valuable information for interventions to sustain teacher well-being.

## Introduction

Over the past two decades, teacher burnout, which has negative impacts on teachers’ well-being, has gained increasing attention from researchers (Cenkseven-Onder and Sari, 2009; Laura et al., 2015). Based on the job demands-resources (JD-R) model of burnout, job and personal demands can lead to exhaustion and the reduction of energetic resources, but the negative impacts can be alleviated by job and personal resources (Demerouti et al., 2001; Bakker et al., 2004; Bakker and Demerouti, 2017). Teaching is an emotionally demanding profession that requires teachers to perform a lot of emotional labor (EL). A growing body of literature supports the influence of EL on emotional exhaustion (EE), which is a core dimension of burnout (Yılmaz et al., 2015), while different emotional labor strategies play different roles: surface acting (SA) as a personal demand and deep acting (DA) as a personal resource (Yin et al., 2018). Yet, how the different EL strategies operate in the straining process of the JD-R model in the educational field is ambiguous.

As defined by Hochschild (1983), EL refers to the management of an employee’s emotions to comply with organizational or occupational emotional display rules. Managing emotions can be accomplished by three main strategies: SA, DA, and expression of naturally felt emotions (ENFE; Hochschild, 1983; Ashforth and Humphrey, 1993; Diefendorff et al., 2005). Surface acting is the act of employees hiding their real feelings or expressing fake emotions to meet display rules without changing their inner states (e.g., Morris and Feldman, 1996). For example, a teacher may hide negative emotions or fake a smile when feeling angry or sad while interacting with students. In deep acting, employees try to actually experience the required emotion and change their inner feelings. For example, a teacher may reappraise a negative emotional experience (e.g., due to a student’s mistake) to display a positive emotional expression. ENFE refers to the kind of strategy in which individuals reflect their emotions as they feel them and the emotions are appropriate for the job. For instance, a teacher who feels angry because of students’ misbehavior may present the emotion to let students reflect on their mistakes.

Previous studies found that emotional labor is significantly related to teachers’ EE, which is the key component of burnout syndrome (Maslach and Jackson, 1981). EE refers to the feeling of physical and emotional fatigue because of being overloaded at work (Yılmaz et al., 2015). Different EL strategies were found to have different effects on teachers’ EE: SA was positively correlated with EE, while DA and ENFE were negatively correlated with EE (e.g., Zhang and Zhu, 2008; Basim et al., 2013; Ye and Chen, 2015; Yılmaz et al., 2015; Yin et al., 2019). Thus, DA and ENFE can be characterized as health-beneficial, while SA is often regarded as health-detrimental (Philipp and Schüpbach, 2010).

There has been a growing amount of research into the emotional spillover between work and family, with more employees engaged in a dual-earner lifestyle (Beutell, 1985; Yanchus et al., 2010; Sanz-Vergel et al., 2012; Rubio et al., 2015; Gu et al., 2019). Thus, the relationships of EL, EE, and work-family conflict (WFC), which is the negative spillover from work to family, have received much attention. WFC, as an aspect of the job or personal demands, was found to be positively associated with EE (Boles and Hair, 1997; Rubio et al., 2015; Rajendran et al., 2020). In addition, a few studies identified a mediating role of WFC in the EL-job burnout (notably EE) relationship (Montgomery et al., 2006; Seery et al., 2008; Noor and Zainuddin, 2011). These findings can enrich our understanding of the intervening role of WFC in the JD-R model among teachers (Liu and Cheung, 2015) or other professions (Sophie et al., 2016). However, to our knowledge, little research has directly investigated the different mediator roles of WFC between various EL strategies (especially ENFE) and EE in the educational field, especially in the Chinese cultural context.

Although the validity of JD-R model has been demonstrated in cross-cultural samples, some researchers have suggested that the impact of culture on JD-R theory should be considered (Gyrks et al., 2012; Rattrie et al., 2020). Hobfoll et al. (2018) argued that “the major rules governing how we respond to stress are embedded within shared cultural beliefs.” The rules of conservation in the stress-strain process in different cultures are the same, but the demands or resources and the ranking of those demands or resources might be different (Gyrks et al., 2012; Hobfoll et al., 2018). Most of the research exploring the impact of culture has consisted of cross-cultural studies comparing samples from different countries, namely, the nation paradigm (e.g., Brough et al., 2013). In consideration of the cultural diversity within countries, a cultural paradigm that used “culture values” as the independent variable is more suitable for analyzing cross-cultural effects (Sawang et al., 2006; Richter et al., 2016). Meanwhile, research directly exploring the cultural effect in the JD-R model has been limited. A meta-analysis explored the moderating impact of national cultural values (i.e., power distance, masculinity/femininity, uncertainty avoidance, long-term/short-term orientation, and individualism/collectivism) on the relationships between job demands/resources and burnout/engagement (Rattrie et al., 2020). However, no study has used cultural values as a potential antecedent for the perception of job and personal demands and resources. Moreover, most previous studies used universal dimensional frameworks of national culture, such as individualism/collectivism (Hofstede, 1980), failing to capture the uniqueness of cultures. The current study examined the culture effect from the insider’s perspective by exploring the impact of the Confucian familism that is at the core of Chinese culture in the JD-R model.

Confucianism has exerted a great influence on Chinese thinking and behavior (Xiong and Wei, 2020). Confucian values relate to individual job attitudes and performance (Zhang et al., 2012; Leong et al., 2014). Confucian familism, as the key aspect of Confucianism, emphasizes the obligations of family members to the family (Yang, 2004). The purpose of this study was to examine the influence of Confucian familism on EE through emotional labor strategies and WFC among Chinese teachers. More specifically, this research was designed to answer the following two questions: (1) Does Confucian familism influence Chinese teachers’ emotional labor strategies, WFC, and EE? (2) Does the WFC of Chinese teachers mediate the relationship between various emotional labor strategies and EE?

## Literature and Hypotheses

### Emotional Labor, Work-Family Conflict, and Emotional Exhaustion

Brotheridge and Lee (2002) used the conservation of resources theory of stress (Hobfoll, 1989) to explain the link between EL and burnout. In response to emotional demands (i.e., occupational emotional display rules), employees expend resources to perform surface and deep acting. EL could result in positive or negative outcomes (gains or loss of resources) in return. When the resources cannot meet the emotional demands, exhaustion follows.

Work-family conflict is defined as “a form of interrole conflict in which the role pressures from the work and family domains are mutually incompatible in some respect” (Greenhaus and Beutell, 1985, p. 77). Time- and strain-based work demands increase WFC because they cause an energy drain and leave individuals unable to meet family responsibilities (Voydanoff, 2004a,b). Liu and Cheung (2015) examined the effects of various work demands and resources on work-family conflict and facilitation in a sample of Chinese secondary school teachers. The study showed that work demands (including emotional demands) were strongly and positively correlated with WFC, which further led to an increase in burnout. Job-related emotional labor, as a reflection of work emotional demands, was found to be associated with WFC because EL taxed employees’ effort and energy in the work domain, the limiting time or energy left for the family domain (Seery et al., 2008). Thus, the emotional labor of teachers at work requires energy and effort, leaving less energy for tasks in the family. Because work and family both require high degrees of emotion management, teachers will experience high WFC, which may eventually reduce resources and increase their emotional exhaustion.

Previous studies found that WFC mediated the relationship between SA and burnout or EE (Montgomery et al., 2006; Seery et al., 2008; Noor and Zainuddin, 2011). Noor and Zainuddin (2011) examined the relationship between EL (including both surface and deep acting), WFC, and burnout in a sample of female Malay teachers with family responsibilities (married, with at least one child at home). They found that WFC only mediated the relationship between SA and EE.

Work-family conflict mediated the relationship between EL and EE, but different EL strategies had different effects on WFC. A few studies found that SA was positively correlated with WFC (Montgomery et al., 2005, 2006; Yanchus et al., 2010; Carlson et al., 2012). Meanwhile, the results for DA have not been consistent: Some studies found that DA was not related to WFC (Montgomery et al., 2006; Seery et al., 2008; Noor and Zainuddin, 2011) or even reduced WFC over time (Gu et al., 2019), yet Karim and Weisz (2013) found that DA was positively correlated with WFC.

Considering the characteristics of different EL strategies, it is not surprising to find a weaker relationship between DA or ENFE and WFC than that of SA. SA expends more emotional and cognitive resources than DA because of the suppression of emotions, although they both require investment of resources (Gross and John, 1997; Richards and Gross, 2000; Grandey, 2003; Yılmaz et al., 2015). ENFE expends the least effort, viewed as “automatic emotion regulation” (Ashforth and Humphrey, 1993; Zapf, 2002, p. 243). In ENFE, individuals only need to make a conscious effort to judge whether their emotions conform to the emotional display rules (Diefendorff et al., 2005). Moreover, DA and ENFE could bring some gain in resources (Brotheridge and Lee, 2002; Yin et al., 2017). Owing to DA’s acquisition and consumption of resources at the same time, DA may have a weaker relationship with WFC than SA, even if it was sometimes found not to be significantly correlated with WFC of teachers. Similarly, ENFE, being effortless, may have the weakest positive correlation with WFC. However, previous studies have not analyzed whether WFC has different mediating roles between various EL strategies (especially ENFE) and EE among teachers. In the present research, we pose the following hypothesis:

*H1*: WFC mediates the relationship between EL and EE. SA is more strongly positively related to WFC than ENFE and DA.

### The Effect of Familism

The emotions of teachers are regarded as not only an individual experience but also a sociocultural experience that conveys sociocultural messages (Zembylas, 2007, p. 61). Therefore, the feeling, expression, and regulation of emotion are influenced by culture. For example, Yin (2016) explored the characteristics of teachers’ EL in the Chinese context and discussed the influence of the Confucian traditional culture. He found that many Chinese teachers could be described by the metaphor of “knife-like mouth and tofu-like heart,” which was due to the emphasis on social order in Chinese traditional culture.

Familism, as an important part of Confucianism, has a great influence on Chinese teachers (Sleziak, 2014). Confucian familism is a strong belief that emphasizes the centrality of the family unit and stresses that people ought to work hard to provide support to family members (Yang, 2004). Influenced by Confucian familism, there are some related ideas in Chinese culture, such as “One’s achievements should be viewed as the family’s achievements” or “Occupational failure brings shame to the family” (Gao et al., 2012, p. S14). Hence, these beliefs push one to work hard and pursue a successful career so that familism may have a positive impact on one’s work performance. In Yang and Cheng’s (1988) study, the Confucian familism of employees in Taiwan’s manufacturing and service industries was positively related to their job performance. Peng (2017) found that Confucian familism was positively correlated with coping styles among Chinese primary school teachers. Therefore, familism may make teachers use more effective emotional labor strategies to achieve their professional goals. SA will lead to resource depletion, which is considered a work-inefficient strategy (Goldberg and Grandey, 2007; Grandey and Sayre, 2019); DA and ENFE can be regarded as work-efficient strategies because of their positive impact on resource acquisition, such as facilitating teacher efficacy (Yin et al., 2017) and a sense of authenticity (Brotheridge and Lee, 2002). This suggests the following hypothesis:

*H2*: Teachers with stronger familism beliefs use more work-efficient EL strategies (i.e., DA and ENFE) and less SA.

Some researchers have suggested that familism is a protective factor that strengthens the emotional connection with family members and provides familial social support in both Chinese (Hwang, 1999; Yang, 2003, 2004) and Latino cultural contexts (Germán et al., 2008; Almeida et al., 2009; Ojeda and Piña-Watson, 2013). However, the negative influence of familism cannot be ignored (Kim et al., 2007; Cucciare et al., 2010; Toyokawa and Toyokawa, 2019), including the effect on well-being and WFC. As Youn et al. (1999, p. 363) pointed out, “familism emphasizes obligation over reciprocal affective ties does not protect against distress and may increase it.” Sun and Pan (2008) found a different result from the Western background in that older Chinese manufacturing workers were more vulnerable to EE than younger ones, because older workers perceived more responsibility to support their family, making them more worried about job stability, lack of training, and delayed pay. Korean caregivers with stronger Confucian familism showed higher levels of depression and anxiety than white American caregivers (Youn et al., 1999). Meanwhile, Hispanic family caregivers with strong familism beliefs were more reactive to the daily occurrence of care-related family disagreement (Koerner and Shirai, 2012).

Consequently, Chinese teachers with a high level of familism may experience more work-family conflict because of their high expectations of positive family involvement, which in turn leads to worse outcomes. However, previous studies on the influence of familism on WFC were mainly conducted in Latino samples (Gelder, 2012) or using the nation paradigm to compare WFC between Confucian Asian countries and Western countries. The findings were not consistent because of the controversial paradigm and the different cultural backgrounds of the samples (Yang, 2005; Spector et al., 2007; Jin et al., 2013). To solve this problem, this study adopts the cultural paradigm to examine the impact of familism on WFC and EE in the context of Chinese culture. The following hypothesis is proposed:

*H3*: Familism is positively related to WFC and EE.

Accordingly, in the Chinese cultural context, this study aims to examine how Confucian familism, EL, and WFC contribute to explaining variance in EE by focusing on the mediating role of EL and WFC. We developed a multivariate model to examine our hypotheses. Based on the previous studies, we hypothesize:

*H4*: EL and WFC sequentially mediate the relationship between familism and EE.

## Materials and Methods

### Participants and Procedure

This research was approved by the Research Ethics Committee of the School of Education of Capital Normal University. Before data collection, we informed the participants about the objectives of this research and the confidentiality of data and confirmed that all data would only be accessible to the research group and used for research purposes. All data in the present study were collected anonymously.

A total of 3,312 Chinese teachers from K-12 schools in Beijing, China, participated in the study (81.9% were female). We distributed questionnaires to teachers through the teacher training department of the municipal Ministry of Education in November 2020. All participants volunteered to participate and completed the questionnaire online. The online questionnaires were administered using Survey Star. The entire procedure lasted approximately 5–10min.

The years of the teaching experience ranged from 0 (new teachers) to 39years (M=17.29, SD=10.04), and 1.9% (63) of the participants did not report this information. Table 1 presents the sociodemographic characteristics of the sample.

**Table 1:** Sociodemographic characteristics of the sample.

		Number	Percentage
Gender	Male	598	18.1%
	Female	2,714	81.9%
Level of education	Vocational education	1,285	38.80%
	Undergraduate education	1,528	46.10%
	Master education	490	14.80%
	Doctor education	9	0.30%
Marital status	Married	2,818	85.10%
	Single	494	14.90%
Monthly income	Under 5,000 yuan	96	2.90%
	5,000–7,000 yuan	580	17.50%
	7,000–10,000 yuan	1,917	57.90%
	Above 10,000 yuan	719	21.70%
Age	Under age 25	219	6.6%
	26–30years old	397	12%
	31–40years old	1,019	30.8%
	41–50years old	1,325	40%
	51–60years old	352	10.6%
School type	Primary schools	1,801	54.4%
	Middle schools	1,000	30.2%
	High schools	511	15.5%

### Measures

#### Confucian Familism

We assessed Confucian familism using the familism subscale from the Confucian Traditional Values Scale developed by Yang (Yang and Cheng, 1988; Yang, 2004). The familism subscale included 11 items. Examples of items include “family members support each other,” “filial devotion to parents,” and “proficiency in a particular line of work.” Participants rated each item using a 4-point Likert scale (1=never important, 4=very important). Cronbach’s *α* for this subscale (11 items) was 0.96.

#### Emotional Labor Strategies

We used the Questionnaire on Emotional Labor developed by Diefendorff et al. (2005), which measures three emotional labor strategies: (1) surface acting (seven items, e.g., “I put on an act in order to deal with customers in an appropriate way”), (2) deep acting (four items, e.g., “I try to actually experience the emotions that I must show to customers”), and (3) ENFE (three items, e.g., “The emotions I express to customers are genuine”). This questionnaire contained 14 5-point Likert-type items (1=strongly disagree, 5=strongly agree). Considering the different interactive partners working with teachers, we replaced “customers” in the original questionnaire with three types of interactive partners (i.e., students, parents, and colleagues and leaders). We calculated the average score of teachers’ emotional labor strategies (i.e., SA, DA, and ENFE) for students, parents, and colleagues and leaders. Cronbach’s *α*s for each subscale were 0.86 (SA for students), 0.80 (DA for students), and 0.91 (ENFE for students); 0.91 (SA for parents), 0.83 (DA for students), and 0.93 (ENFE for parents); 0.94 (SA for colleagues and leaders), 0.82 (DA for colleagues and leaders), and 0.94 (ENFE for colleagues and leaders).

To ensure the consistency of Chinese and English versions, the items were translated into Chinese by two translators and were then back-translated by other translators.

#### Emotional Exhaustion

The teachers’ emotional exhaustion was measured with the emotional exhaustion subscale from a Chinese version of the Maslach Burnout Inventory for Educators which has been revised and published in China (Wu et al., 2016). The subscale included eight items (e.g., “I feel exhaustion at the end of workday”). Responses were made on a 5-point Likert-type scale. Cronbach’s *α* for this subscale was 0.96.

#### Work-Family Conflict

The WFC Scale was developed by Carlson et al. (2000) and has been translated and used in China (Yu, 2006). This scale consisted of nine 5-point Likert-type items to measure the work-to-family direction of conflict. The scale has three factors: (1) time-based WFC, which occurs when time devoted to work makes it difficult to participate in family activities (three items, e.g., “My work keeps me from my family activities more than I would like”); (2) strain-based WFC, which suggests that strain experienced at work intrudes on and interferes with participation in family (three items, e.g., “I am often so emotionally drained when I get home from work that it prevents me from contributing to my family”); and (3) behavior-based WFC, which occurs when specific behaviors required at work are incompatible with behavioral expectation in the family (three items, e.g., “behavior that is effective and necessary for me at work would be counterproductive at home”). The Cronbach *α* for this scale was 0.94 (time-based WFC: *α*=0.93; strain-based WFC: *α*=0.93; behavior-based WFC: *α*=0.89).

### Data Analysis

The internal consistency, descriptive statistics, and correlations among the variables were analyzed using SPSS 18.0. Full information maximum likelihood procedures were applied to handle the missing data. According to the recommendation of Preacher and Hayes (2004, 2008), we tested the multiple mediation models with Mplus 8.0 and used a bootstrapping procedure to examine the direct and indirect effects as well as the significance of the mediating effects. An indirect effect was considered significant if the 95% bias-corrected confidence interval did not overlap with zero. Following the suggestions of previous studies (e.g., Hu and Bentler, 1999; Schreiber et al., 2006), we used the following indices to evaluate model fit: root mean square error of approximation (RMSEA)<0.08, standard root mean square residual (SRMR)<0.10, comparative fit index (CFI)>0.90, and Tucker-Lewis index (TLI)>0.90.

## Results

### Descriptive Statistics

The descriptive statistics and correlations between the variables are presented in Table 2. These results provide a foundation for mediation analysis. As expected, familism, SA, DA, ENFE, WFC, and EE were significantly correlated with each other. Among them, familism was positively correlated with DA, ENFE, WFC, and EE and negatively correlated with SA; EE was positively correlated with DA, SA, and WFC and negatively correlated with ENFE; WFC was positively correlated with SA, DA, and ENFE. Additionally, years of teaching experience were found to be positively correlated with ENFE and EE and negatively correlated with SA. Therefore, years of teaching experience were put into the subsequent model as a control variable because it correlated with the outcome variables.

**Table 2:** Descriptive statistics and correlations for the variables in this study.

S. No.		M	SD	1	2	3	4	5	6	7
1.	Years of teaching experience	17.29	10.04	1.00						
2.	SA	2.77	0.82	−0.13^***^	1.00					
3.	DA	3.59	0.67	0.02	0.35^***^	1.00				
4.	ENFE	3.85	0.70	0.10^***^	−0.27^***^	0.45^***^	1.00			
5.	WFC	3.48	0.90	0.02	0.35^***^	0.24^***^	0.04^*^	1.00		
6.	EE	3.59	0.97	0.14^***^	0.28^***^	0.12^***^	−0.06^***^	0.61^***^	1.00	
7.	Familism	3.61	0.51	0.05^*^	−0.10^***^	0.25^***^	0.33^***^	0.08^***^	0.10^***^	1.00

* *p<0.05;*

*** *p<0.001*.

### Measurement Model

We first evaluated the measurement model to assess whether latent variables were well-represented by indicator variables using Mplus 8.0. The confirmatory factor analysis was conducted with 6 latent factors and 31 observed variables. The latent variable familism was indexed by 11 indicators (11 items from the familism subscale of the Confucian Traditional Values Scale). The latent variable EE was indexed by eight indicators (eight items). The latent variable SA was indexed by three indicators (SA for students, SA for parents, SA for colleagues, and leaders). The latent variable DA was indexed by three indicators (DA for students, DA for parents, and DA for colleagues and leaders). The latent variable ENFE was indexed by three indicators (ENFE for students, ENFE for parents, and ENFE for colleagues and leaders). The latent variable WFC was indexed by three indicators (time-based WFC, strain-based WFC, and behavior-based WFC). The estimation of the measurement model revealed a satisfactory fit to the data: *χ*^2^=6475.64, *df*=419, *RMSEA*=0.07, *TLI*=0.93, *SRMR*=0.03, *CFI*=0.93. All the factor loadings for the indicators on the latent variables were significant (*p*s<0.01), and the standardized factor loadings ranged from 0.66 to 0.95, indicating that all the latent factors were well-represented by their respective indicators. All the latent factors from the measurement model were significantly correlated (*p*s<0.01).

### Hypothesis Testing

**Figure 1:**
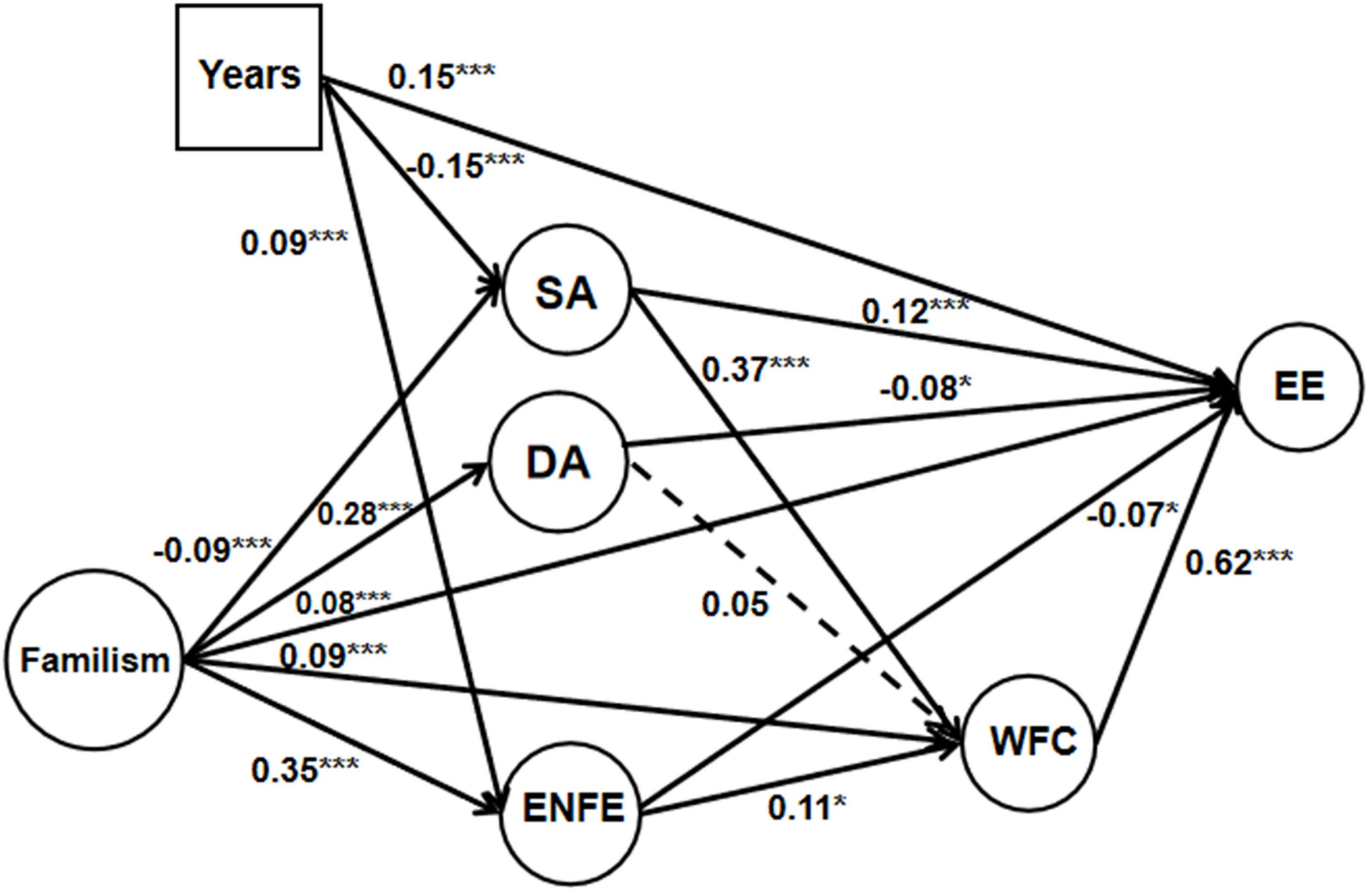
The relationships between familism, EL, WFC, and EE, controlling for years of teaching experience (the solid line represents the significant path; the dotted line represents the insignificant path). ^*^p<0.05; ^***^p<0.001.

We applied structural equation modeling using Mplus 8.0 and the maximum likelihood estimation method to examine the hypothesized model. In the model, emotional labor strategies (SA, DA, and ENFE) and WFC sequentially mediated the effect of familism on EE after controlling for years of teaching experience (Figure 1). Because the correlations between years of teaching experience and DA/WFC were not significant (*p*s>0.05), we only controlled the effect of years of teaching experience among SA, ENFE, and EE (*p*s<0.001).

The model revealed a good fit to the data (*χ*^2^=6710.261, *df*=446, *RMSEA*=0.065, *TLI*=0.93, *SRMR*=0.03, *CFI*=0.93). However, the standardized path coefficient from DA to WFC was non-significant (*ß*=0.06, *p*=0.39), and all other path coefficients were significant (Figure 1). Specifically, familism positively predicted DA, ENFE, WFC, and EE and negatively predicted SA, supporting H2. SA and ENFE had significant positive effects on WFC. ENFE negatively predicted EE, while WFC and SA positively predicted EE.

We generated 5,000 bootstrapping samples from the original data *via* random sampling to examine the mediation effects (Preacher and Hayes, 2008). Zhao et al. (2010) identified the different patterns of mediation: The mediation is complementary if the indirect effect and direct effect both exist and point in the same direction; the mediation is competitive if the indirect effect and direct effect both exist and point in opposite directions; it is indirect-only mediation if there is no significant direct effect, but the indirect effect is significant, which was called full mediation by Baron and Kenny (1986).

As shown in Table 3 and Figure 1, the results suggested that our hypotheses were all supported. First, the indirect effects of SA and ENFE on EE *via* WFC were significant and positive (SA: *ß*=0.23, *p*<0.001; ENFE: *ß*=0.07, *p*<0.05), supporting H1, and the indirect effect of SA was greater than that of ENFE. In addition, the direct effect of SA on EE was significant and positive (*ß*=0.12, *p*<0.001), so the mediation was complementary. However, the mediation of WFC between ENFE and EE was competitive because of the negative direct effects of ENFE on EE (*ß*=−0.07, *p*<0.05).

**Table 3:** Direct and indirect effects and 95% confidence intervals in the test model.

Model pathways	Estimated standardized effect	BC 95% CI	*p*
***Total effect***			
Familism→EE	0.090	[0.052, 0.127]	0.000
Familism→WFC	0.111	[0.074, 0.149]	0.000
***Direct effects***			
SA→EE	0.122	[0.057, 0.187]	0.000
ENFE→EE	−0.073	[−0.142, −0.002]	0.043
DA→EE	−0.075	[−0.149, −0.005]	0.045
Familism→EE	0.078	[0.044, 0.113]	0.000
Familism→WFC	0.094	[0.059, 0.130]	0.000
***Indirect effects***			
SA→WFC→EE	0.231	[0.181, 0.273]	0.000
ENFE→WFC→EE	0.070	[0.017, 0.128]	0.012
DA→WFC→EE	0.028	[−0.035, 0.092]	0.386
Familism→SA→EE	−0.011	[−0.020, −0.005]	0.002
Familism→DA→EE	−0.020	[−0.042, 0.002]	0.049
Familism→ENFE→EE	−0.025	[−0.050, −0.001]	0.044
Familism→WFC→EE	0.059	[0.037, 0.080]	0.000
Familism→SA→WFC→EE	−0.022	[−0.033, −0.013]	0.000
Familism→DA→WFC→EE	0.008	[−0.010, 0.025]	0.388
Familism→ENFE→WFC→EE	0.024	[0.006, 0.045]	0.014
Familism→SA→WFC	−0.035	[−0.052,−0.020]	0.000
Familism→DA→WFC	0.012	[−0.016, 0.041]	0.386
Familism→ENFE→WFC	0.039	[0.010, 0.073]	0.014

Second, the total and direct effects of familism on EE were significant and positive (total: *ß*=0.090, *p*<0.001; direct: *ß*=0.078, *p*<0.001), confirming H4. We added up all the positive and negative significant indirect effects of familism on EE separately. The total positive indirect effect was 0.083, and the total negative indirect effect was −0.078. The total positive effect (include indirect and direct) was 0.161.

Third, the link between familism and EE was sequentially mediated by SA and WFC (SA: *ß*=−0.022, *p*<0.001). ENFE and WFC also sequentially mediated the relationship between familism and EE (ENFE: *ß*=0.024, *p*<0.05). Moreover, this sequential mediation of SA and WFC was competitive, while the mediation of ENFE and WFC was complementary. We also found that there were competitive mediations of both SA and ENFE between familism and EE, and there was a complementary mediation of WFC between familism and EE.

Fourth, the total and direct effects of familism on WFC were notable and positive (total: *ß*=0.111, *p*<0.001; direct: *ß*=0.094, *p*<0.001), confirming H3. The total positive indirect effect of familism on WFC was 0.039, and the total negative indirect effect was −0.035. The total positive effect (including indirect and direct effects) was 0.133.

Furthermore, we also found that there was a competitive mediation of SA between familism and WFC, and there was a complementary mediation of ENFE between familism and WFC.

## Discussion

Most studies concerning the relationship between antecedents and job stress have focused more on job or personal demands and less on the influence of the wider social-cultural background. Integrating job, personal, and cultural factors into the JD-R model, the current study attempted to obtain better insights into the possible associations between familism, EL, WFC, and EE, with special attention to the possible mediating role of EL and WFC among Chinese teachers. The results of multiple mediation models were consistent with our hypotheses except the relationship between DA and WFC. This research provides valuable information to help understand the intervening roles of WFC and EL and the cultural effect in the JD-R model. Our findings are valuable to both the development of the JD-R theory and its application in education.

### Theoretical Implications

#### Emotional Labor, Work-Family Conflict, and Emotional Exhaustion

The results of the current study support our hypothesis that WFC has different mediating roles between various EL strategies (i.e., SA, DA, and ENFE) and EE among teachers.

First, the results revealed that SA was significantly related to the EE of teachers, while DA and ENFE were negatively related to the EE of teachers. These results are consistent with many previous studies in various cultural contexts (e.g., Zhang and Zhu, 2008; Philipp and Schüpbach, 2010; Basim et al., 2013; Ye and Chen, 2015; Yin et al., 2019). We also found that SA had a stronger impact on EE than DA and ENFE, in agreement with Yao et al. (2015). Our findings suggested that DA and ENFE could benefit the conservation of resources (Brotheridge and Lee, 2002; Yin et al., 2017), while the resource gain through DA or ENFE was lower than the resource loss through SA (Brotheridge and Lee, 2002). This is consistent with the dual pathways in JD-R theory: Demands are the most important predictors of burnout, and resources are the most important predictors of motivation (Bakker and Demerouti, 2017).

Second, in line with previous research (Montgomery et al., 2005, 2006; Yanchus et al., 2010; Carlson et al., 2012), our findings showed that WFC was positively related to SA and not significantly related to DA. Consistent with our hypothesis, the results revealed that SA had the greatest impact on WFC. Surprisingly, however, ENFE had a greater positive impact than DA, owing to the use of EL strategy in family. The use of EL strategies at work was found to be positively related to the use of EL strategies at home (Liu et al., 2018). That is, the more DA teachers used at work, the more DA they used at home. When facing family members, trying to adjust their cognition to regulate emotions may help teachers solve family conflicts better. Thus, DA is not a bad strategy to use at home, while using SA and ENFE at home may have negative effects on relationships with family members. Liu et al. (2018) found that surface acting at home mediated the relationship between surface acting at work and spousal ratings of family quality, while DA at home was not related to family quality. In ENFE, Chinese teachers could express negative emotions, such as by “scolding them or using sharp words,” to help students understand them and achieve their teaching goals (Yin, 2016, p. 15). However, venting negative emotions when facing family members will lead to worse consequences. Therefore, ENFE may cause more WFC than DA.

Third, we also found that WFC mediated the relationship between SA and EE as well as between ENFE and EE. These results are consistent with previous studies and add to a growing body of evidence that work and family spill over into each other through emotion (Montgomery et al., 2006; Seery et al., 2008; Noor and Zainuddin, 2011; Sanz-Vergel et al., 2012). In addition, the results showed that the mediation of WFC between SA and EE was complementary, but the mediation between ENFE and EE was competitive. These findings suggested that WFC has different mediating roles between various EL strategies and EE among teachers: Both the direct and indirect effects of SA on EE were positive, while there was a negative direct effect of ENFE on EE and a positive indirect effect of ENFE on EE that was mediated by WFC. This competitive mediation indicated that ENFE plays a dual role in EE. On the one hand, ENFE at work may bring work-related resources to reduce EE; on the other hand, it will spill over into family and lead to EE by increasing WFC.

#### The Effect of Familism

By examining the influence of familism on EL, WFC, and EE, we identified significant effects of cultural factors in a JD-R model from an insider’s perspective. Most studies in the field of cultural impact have only focused on the moderating role of culture and used universal cultural dimensions that attempt to describe differences between cultures. Such approaches, however, have failed to address the uniqueness and complexity of cultures. Our results provide further insights into the specificities and nuances of Chinese culture and their effects on work-related outcomes.

Our findings showed that familism had positive impact on DA and ENFE and a negative impact on SA, supporting H2. Chinese teachers influenced by Confucian familism are motivated to use less SA and more DA and ENFE strategies. These results reveal the influence of cultural factors on personal variables (i.e., demands and resources), which is consistent with the positive effect of familism on work performance found in previous studies (e.g., Yang and Cheng, 1988; Peng, 2017).

Moreover, the results showed that familism was positively associated with the WFC of teachers, confirming H3. It suggested that the emphasis on familism made them more likely to experience work-family conflict. In contrast to our results, mixed evidence regarding the differences in WFC between individualistic countries and collectivistic Confucian Asian countries was previously reported: Some studies found no significant differences, while some studies found more WFC in individualistic countries (e.g., North America) than in collectivistic countries (e.g., China), or vice versa (Yang, 2005; Spector et al., 2007; Jin et al., 2013). This inconsistency in results may be due to the different paradigms used. These cross-cultural studies adopting the nation paradigm used “country” as the independent variable and interchangeably with cultural values. However, the effects of country and cultural values on the variables within stress and the coping process are different (Sawang et al., 2006). This study used the cultural paradigm to provide more evidence about the relationship between familism and WFC, revealing the influence of culture on the perception of job demands.

Our results deepened the understanding of the differences in WFC between collectivist and individualist countries that have appeared in previous studies. Eastern collectivist countries, such as China, are influenced by the Confucian culture and attach great importance to family obligations. Family members are mutually dependent and closely connected. Chinese employees even regard work as a means to improve the family’s economic well-being and fulfill family responsibilities (Aryee et al., 1999). On the one hand, they have high expectations of family involvement. On the other hand, they need to work hard to support their families. In this way, the centrality of the family and the interdependence among family members can cause considerable WFC and pressure. In some individualist countries or areas, on the contrary, people have fewer family demands and pay more attention to personal interests (Mortazavi et al., 2009). For example, Aryee et al. (1999) found that the life satisfaction of Hong Kong employees was primarily influenced by WFC, while that of American employees was mainly influenced by family-work conflict. Previous studies have shown that the country-level variables have a relatively small direct effect on WFC (Masuda et al., 2019). Our results indicated that familism may be helpful in explaining work-family relations in the Confucian culture.

Finally, and more importantly, this study elucidated the mechanisms underlying the relationship between familism and EE by introducing EL (i.e., SA, DA, and ENFE) and WFC into the model, and familism had both a positive and negative impact on EE. These findings contribute to the JD-R theory by revealing the antecedent roles of culture and further support and enrich the JD-R theory in the Chinese context. Our findings showed that WFC and EL mediated the relationship between familism and EE among teachers: The direct effect was positive, while there were both positive and negative indirect effects. Our findings indicated that these Chinese teachers influenced by Confucian familism tended to use more work-efficient EL strategies so as to reduce EE. However, teachers with strong familism beliefs would perceive more WFC and eventually increase their EE. These findings suggest that Confucian familism may play the dual role of motivator and stressor for Chinese teachers’ emotional labor and well-being. On the one hand, Confucian familism as a cultural demand can force teachers to invest effort in their work, which leads to physical and psychological costs. On the other hand, as a cultural resource, it can also drive teachers to achieve work goals, improve work performance, and reduce the associated physiological and psychological costs (Bakker and Demerouti, 2017).

### Limitations and Future Research

The implications of this study should be interpreted in light of its limitations. First, our study used a cross-sectional design, so we could not make any causal inferences. A longitudinal design in future research will yield more solid causal relationship evidence. Second, although we found a positive total effect of familism on EE, it is hard to be sure that familism is more of a pressure than a motivator. These results must be interpreted with caution because there may be other factors mediating the relationship between familism and EE, such as family support and family-to-work enrichment, which may have negative effects on EE (Liu and Cheung, 2015). Accordingly, we recommend that future studies examine whether there exist other possible negative indirect effects of familism on EE.

### Practical Implications

In view of the general need to promote the well-being of teachers, it is essential to explore the factors influencing emotional exhaustion and their roles in the health impairment process. From a practical perspective, our findings suggest that DA is a health-beneficial strategy that teachers should use more, while SA is a health-detrimental strategy that teachers should use less. In addition, ENFE is a double-edged sword considering its contrasting impacts on WFC and EE, so teachers need to use this strategy carefully. Considering that teachers with more years of teaching experience will use SA less, schools can invite experienced teachers to help younger teachers learn emotion management.

Moreover, given that EL can increase WFC, training and interventions for teachers need to pay attention to the contents of conflict management and relief. The finding that DA was not related to WFC suggests that training programs should help teachers understand the nature of DA and its applications in the teaching profession.

Last but not least, cultural background must be taken into account in interventions in teacher burnout and teacher training. Chinese schools should consider the influence of Confucian culture when making policies and raise awareness of teachers’ needs to fulfill family responsibilities to ensure that teachers can have some balance between their work and family demands. Furthermore, the dual role of familism reveals the complexity of cultural effects, suggesting that teacher training should help teachers think dialectically about traditional values. According to our findings, the promotion of teacher well-being should not only rely on teachers’ self-regulation but should also be supported by the society, school, and family.

## Data Availability Statement

The raw data supporting the conclusions of this article will be made available by the authors, without undue reservation.

## Ethics Statement

The studies involving human participants were reviewed and approved by Research Ethics Committee of the School of Education of Capital Normal University. Written informed consent for participation was not required for this study in accordance with the national legislation and the institutional requirements.

## Author Contributions

XZ, GT, HY, and WH designed the study. GT and XZ collected and analyzed data. XZ, GT, and HY wrote the manuscript. HY reviewed and revised the manuscript. All authors contributed to the article and approved the submitted version.

## Conflict of Interest

The authors declare that the research was conducted in the absence of any commercial or financial relationships that could be construed as a potential conflict of interest.

## Publisher’s Note

All claims expressed in this article are solely those of the authors and do not necessarily represent those of their affiliated organizations, or those of the publisher, the editors and the reviewers. Any product that may be evaluated in this article, or claim that may be made by its manufacturer, is not guaranteed or endorsed by the publisher.
